# Stable and robust development of orientation maps and receptive fields

**DOI:** 10.1186/1471-2202-12-S1-P10

**Published:** 2011-07-18

**Authors:** Judith S Law, Jan Antolik, James A Bednar

**Affiliations:** 1 Institute for Adaptive and Neural Computation, University of Edinburgh, Scotland, UK; 2 Unité de Neurosciences Information et Complexité, CNRS, Gif-sur-Yvette, France

## 

**Figure 1:**
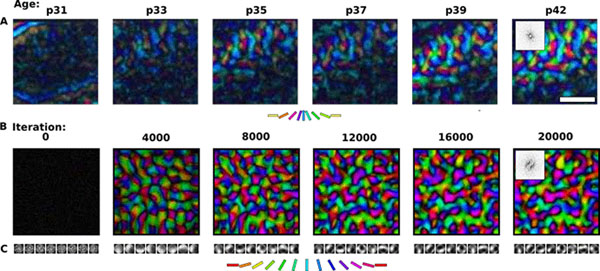
(A) Polar orientation maps recorded in a ferret using chronic optical imaging at the postnatal ages shown (in days; reprinted from [1]). (B) GCAL model polar orientation maps. Spontaneous activity patterns drive the map development until 6000 image presentation iterations, after which natural images are presented to the model retina. (C) Patterns of connectivity from the LGN to 7 arbitrarily selected V1 neurons, showing how orientation selectivity emerges over time. Both the ferret and model map have a ring shaped FFT (inset in the final map plot of A and B).

Development of orientation maps in ferret and cat primary visual cortex (V1) has been shown to be *stable*, in that the earliest measurable maps are similar in form to the eventual adult map [1], *robust*, in that similar maps develop in both dark rearing and in a variety of normal visual environments [2], and yet *adaptive*, in that the final map pattern reflects the statistics of the specific visual environment [3]. How can these three properties be reconciled? Using a mechanistic model of the development of neural connectivity in V1, we show how including two low-level mechanisms originally motivated from single-neuron results makes development stable, robust, and adaptive. Specifically, contrast gain control in the retinal ganglion cells and the LGN reduces variation in the pre-synaptic drive due to differences in input patterns, while homeostatic plasticity of V1 neuron excitability reduces the post-synaptic variability in firing rates. Together these two mechanisms lead to maps that develop stably and robustly, yet adapt to the visual environment. The modeling results suggest that topographic map stability is a natural outcome of low-level processes of adaptation and normalization. The resulting GCAL model is also significantly simpler yet more robust and more biologically plausible than previous mechanistic models of cortical map, receptive field, and connection development, and thus represents a good platform for future cortical modeling. The simulator and the model code can be freely downloaded from topographica.org. Figure 1.
